# On societal response to pandemics: linking past experiences to present events

**DOI:** 10.1007/s44155-022-00012-2

**Published:** 2022-07-01

**Authors:** Gazala Khan, Sazzad Parwez

**Affiliations:** 1 Department of English, School of Languages, Doon University, Dehradun, Uttrakhand India; 2 School of Liberal Arts and Human Sciences, Auro University, Surat, India

**Keywords:** Pandemic, Historical, Literature, COVID-19 pandemic

## Abstract

There is a need for a factual understanding of the historical impact of pandemics in the world. Against this backdrop, this study provides a historical understanding of societal behaviour and responses to pandemics. Inferences are primarily drawn from a literature review from the past and present. The present analysis also reflects the ongoing COVID-19 pandemic in the world and India while providing a novel perspective to understand public health practices in a global context. It suggests the need for a more synchronised health response in pandemics while highlighting the uncertainties and challenges with historical evidence and comparisons to the ongoing pandemic. An emphasis is on learning from historical evidence and ascertaining how these retrospective diagnoses help make arguments about health and illness in our present moment.

## Introduction

Given the fragmented understanding of pandemics, there is a need for a historical account in the backdrop of the present scenario. COVID-19 is the first global pandemic with extensive print, electronic, and social media documentation capturing both information and misinformation [1]. With uncertainty in the pandemic, people struggle for ideas to effectively communicate in lockdown with artworks, songs, cartoons, and online messages to deal with the loneliness of isolation. Yuval Noah Harari [2] suggested that social distancing is inevitable during the crisis as the virus spreads by exploiting human instincts of socialisation and contact, especially in hard times. The virus is a mindless microbe; we humans can analyse and change how we behave [3].

Over the millennia, pandemics/epidemics have been mass killers on a massive scale. Even today, nearly half a million people are victims of diseases such as Malaria every year [4]. Considering the 6th-century plague of Justinian, which killed possibly half the worldwide population at that time, was a devastating event [5]. The ‘Black Death’ in the fourteenth century may have killed up to 200 million people [6–9]. Further, the twentieth century witnessed the death of as many as 300 million people caused to Smallpox [10].

Pestilence, illness, plague, pandemic, malady, sleeping sickness, and disease: all these terms trace the same path towards a single journey: death. "And Darkness and Decay and the Red Death held illimitable dominion over all" [11]. 2020 and 2021 in the Georgian calendar are well-known catastrophic repercussions of the new coronavirus. The only universal thing is microbes. In the twenty-first century, the world where human beings reigned is taken over by Severe acute respiratory syndrome (SARS-COV-2), also known as coronavirus or COVID-19, claiming thousands of lives every day [4, 12, 13]. The virus does not differentiate between people of different races, nations, ethnicity, colour, caste, and creed.

In history, the world has known and experienced several maladies that hampered the whole ecosystem and led to stark differences in varied populations, thus altering the demography. Whenever a pestilence surfaced in the form of a pandemic, it ravaged the human world; be it Athens Plague, Justinian Plague, Black Death, the Great Plague, Spanish Influenza, Cholera pandemics, or COVID-19, have had changed/altered the demographic ratio [14–16].

Presently, the *Danse Macabre* is part of the world, which means 'Dance of the Death.' during Medieval times, the artistic genre came into foresight in art, music, dance, and drama, where a group of skeletons subpoenaed living human beings from all walks of life to carry the corpse to its deathbed [17]. The primary purpose of the art pieces acted as a *memento mori* representing the inevitability of death. The *Nuremberg Chronicler,* an illustrated encyclopedia of the history of the world compiled in 1493 by Hartmann Schedal, has a mural by Michael Wolgemut titled *Dance of the Death* [17, 18]. The horrendous and un-forgetful incidents of one of the deadliest plagues, i.e. 'The Black Death,' are recorded from medieval times (1000–1500 CE). The French poet Camille Saint-Saëns (1835–1921) wrote a symphonic poem for orchestra music titled *Danse Macabre, the same title used by* Stephen King for a book published in 1981 on the pandemic [3, 17, 19]. It is observed that different art forms have tried to bring into focus the picture of the plagues and pandemics that impacted billions of lives in a short period.

In the history of humankind, COVID-19 is not the first pandemic that has claimed millions of lives. The plague is well documented and has been part of the world since the fifth century, and thrived in dormant forms for centuries. People died of typhus, cholera, tuberculosis, AIDS, etc. These diseases claimed many lives, yet they do not come under the definition of a pandemic.

There had been a few instances of epidemics in the twenty-first century; for example, the country of Sierra Leone, mapped in West Africa, quivered under the pestilence of the Ebola virus. The Ebola Virus was first detected in the Democratic Republic of Congo in 1978 and was declared an epidemic from 2013 to 2016 in West Africa [20]. It is considered an epidemic, whereas COVID-19 is reported as a pandemic [6]. So, what is the difference between an epidemic and a pandemic? In its newsletter, the World Health Organization [21] defines an epidemic as “the occurrence in a community or region of cases of an illness, specific health-related behaviour, or other health-related events clearly above normal expectancy." A pandemic is "an epidemic occurring worldwide, or over an extensive area, crossing international boundaries and usually affecting many people” [21]. The geographical space is the critical factor differentiating an epidemic from a pandemic [17].

Against this backdrop, this study explores the historical understanding of societal behaviour and responses to pandemics. It is essential because there is a lack of factual understanding of the pandemic history and social behaviour, which makes this study critical in the present times. This study also reflects the ongoing COVID-19 pandemic to provide an understanding of societal and public health responses from a global perspective.

## Research methods

This study is a literature-based narrative attempting to capture the complex nuances of people, events, responses, and even knowledge on pandemics of the time that have influenced past behaviour and shaped the present. It examines literature throughout a period that emerged and then traced its evolution within the scholarship of pandemics with illustrations from the ongoing COVID-19 pandemic.

The literature review is interdisciplinary and focuses on history to trace the personal, socio-economic, political, economic, and cultural impacts on the human race that ravage and withered every time a pandemic occurs. The factual understanding is sourced from extensive literature, print and electronic media, narrative accounts, dance, and drama documented over time. This is appropriate to the study and allows the generalisation of findings.

## Pre-scientific revolution

The Plague of Justinian spread like a pandemic from 541 to 542 AD, which claimed millions of lives. It was named after the emperor of the Byzantine Empire, Justinian I, also known as Justinian the Great. Later through the DNA sampling of the bones, it was observed that it was a bubonic plague (Yersinia pestis) [22]. Other forms of plague-like pneumonic and septicemic were also recorded during the pandemic [23]. It took people's lives for more than two hundred years and finally disappeared in 750 AD. The historical account of Procopius of Caesarea, a roman high ranking official who lived in Constantinople, and John of Ephesus, a Christian bishop in Syria, reported a long historical account of the plague [7]. They described the disease as characterising victims with swollen and hardened parts of the body, especially armpits and groin areas. It was marked by oozing of the swollen areas in many cases. The John of Ephesus concluded that it was due to the emperor Justinian's wrongdoings and evil offerings, which angered God, punishing humanity through the plague [14, 23].

The database from the historian Procopius records that the plague spread from the African continent to Mediterranean and European banks through grain transportation. The black rats carried the disease from one place to another, infecting hundreds of thousands. The emperor of the Byzantine empire took unprecedented steps to tackle the situation when the space for burial of the dead bodies became scant; he ordered the emptying of the burial sites and extending the burial of the victims outside the walls of the empire. It can be considered the first pandemic in history. Procopius's description of the plague is recorded in his book *Secret History* [20, 23].

The second and the deadliest pandemic in history is the 'Black Death' of the fourteenth and fifteenth centuries. Geoffrey Chaucer, the father of English literature, was the greatest poet of medieval times. His most significant work *Canterbury Tales* commences referring to the deadliest ‘Black Death’, which claimed millions of lives [24].

The days of the death have passed, and now the pilgrims are on a journey to pay homage to St. Beckett. It begins as:

- 1 Whan that April with his shores soote
       *When April with its sweet-smelling showers*
- 2 The droghte of March hath perced to the roote,
       *Has pierced the drought of March to the root,*
- 3 And bathed every veyne in swich licour
       *And bathed every vein (of the plants) in such liquid*
- 4 Of which vertu engendered is the flour;
       *By the power of which the flower is created;*
- 5 Whan Zephirus eek with his sweet breath
       *When the West Wind also with its sweet breath*. [25]

This prose is about hope in the time of death and dispair and it talks about spring’s renewal and rebirth. The natural world’s reawakening aligns with talks about earth readying itself for spring. It reflects hope being the biggest strength of people in times of crisis.

The Black Death of the fourteenth century dating from 1346 to 1353, was a bubonic plague, a bacterial infection caused by the bacteria *Yersinia pestis* [26]. The pandemic enmeshed the European societies' social, religious, and economic status. The demographic changes were evident as it is noted that the cataclysmic loss of lives in Europe is claimed to be the one-third population of the continent. In addition to this, people lost faith in the church as the supreme entity. In the later centuries, the emergence of science and technology allowed for better treatment scope. The social and cultural milieu were mainly represented through literary and historical anecdotes. Diane Bani-Esraili, in research titled "A New Relationship with Death: A Synthesised Experiential Portrait and Analysis of Mid-Fourteenth Century Medieval European Society's Reception of, Responses to, and Reflections on the Black Death," cites Italian painter and Petrarch views on the number of historical accounts on high fatalities due to the Black Death.

*So graphic and deadly was this plague that the great contemporary Florentine author Francesco Petrarch (1304-1374) legitimately worried that future generations would mistake historical eyewitness accounts of the event for tall tales. In a correspondence with a friend, Petrarch wrote: “O happy posterity, who will not experience such abysmal woe and will look upon our testimony as a fable.” *[27]

The quotation proposes that 'Black Death' is not about symptoms of the plague; rather, it is simply the consequence of a mistranslation. It caused the graphic and painful death of people in Europe. Resultantly, it became the focal point of both contemporary writers and scholars reflecting on the vulnerability of people and society.

Another famous Italian poet Giovanni Boccaccio in his masterpiece *The Decameron* (1349–1353), refers to the situations faced by the people during the plague. The background of the Italian classical prose is in medieval Italy, where seven women and three men take refuge in a deserted village to escape the ‘Black Death. They decide to narrate stories and pass their time. The stories represented medieval society's socio-economic and political amalgamation [28, 29]. The term ‘Black Death’ is comparatively a recent term that came into vogue and then commonly accepted by most nineteenth and twentieth-century social scientists and readers. Earlier it was referred to as pestilence or disease. The term ‘Black Death’ was coined as a result of mistranslation. The Latin term *Atra mors* was used in the historical accounts for naming the pestilence. It means 'terrible death,' but English translations that emerged widely accepted were translated as ‘Black Death’ [7, 24, 27, 30]. There is no connection between the designated term 'Black Death' and the cataclysmic events or the pestilence.

The modern interpretation of the famous nursery rhyme also referred to as a folk song, gives glimpses of how details were passed on through rhymes. The song is:

*Ring o ring o roses*

*a pocket full of posies*

*Achoo! Achoo!*

*And all fall down* [12].

This folk song raises awareness about the symptoms and implications of the ‘Black Death. The context is the patients' symptoms where they develop red coloured rings like roses. Posies were medicinal flowers scrubbed around these rings for treatment; the patients used to keep them in pockets, reflecting on healthcare understanding and fear among people.

There was no medication except for people to quarantine themselves and save their lives by keeping away from the contamination. Quarantine measures and physical distancing have been proved a significant step in controlling the plagues in the pre-scientific temporal frame.

Plague and pestilence were relatively regular tragedies in the ancient world, followed by scary reports [30]. In those times, with a plague spread, no medicine was helpful, and it was unstoppable. The only way to escape the plague was to avoid contact with others. Generally, the plague was considered God’s punishment for misdeeds. On the other hand, the Greek historian contradicted a mystical basis of the disease. Later medieval texts emphasised human behaviour with increased greed and corruption, which caused infection and thus both moral and physical death.

## Post-scientific revolution

The post-scientific period will include the period from the fifteenth century to the present. Science and technology were seen as catalysts for better health-related infrastructure and techniques that brought humans to the centre of the ecosystem. Though better medical facilities came into existence, pandemics still invaded the human homeland. The pestilences agitated the human world again and again. The Great Plague of 1665, The Spanish Flu, Cholera Pandemics, and the COVID-19 Pandemic left a catastrophic and cataclysmic impact on the human world.

A diarist in England, Samuel Pepys, gives a vivid description of the Great Plague of 1665, ravaging many lives and emptying thousands of houses. The Great Plague of London was a bubonic plague that devastated a fifth of the total population of London [23].

Samuel Pepys (1665) says, *"In the evening home to supper, and there to my great trouble hear that the plague is come into the City (though it hath these three or four weeks since its beginning been wholly out of the City); but where it begins but in my good friend and neighbour's, Dr Burnett in Fenchurch Street - which in both points troubles me mightily. To the office to finish my letters, and then home to bed, being troubled at the sickness ... and particularly how to put my things and estate in order, in case it should please God to call me away.”* [23]

It was reasonable quarantine measures that helped the city dwellers to eradicate the plague.

Salman Rushdie says, *“On almost every page of that book ... the way people behaved in the 17th century is exactly how they behave now,… The book details how some Londoners locked down, sequestered themselves and successfully avoided the disease, while others "refused to do that, [saying] it was an intrusion on their freedom — and many of them died," Rushdie said. There were also "quacks peddling crazy cures" and political wrangling around how to handle the outbreak…*

"*It just showed me that we are just who we are, human nature is what it is, and back in 1665, people were doing exactly what people have been doing during this plague year,*"

"*I do not know whether that is comforting or not, but it seems as a species, we have not grown up very much since the 17th century.*" [31]

The Third Plague Pandemic was first reported in Yunnan, China, in 1855. It spread to nearby continents through global trade and voyages through ocean and land. It is considered one of the deadliest pandemics that claimed more than 10 million lives [32]. According to World Health Organization, the pandemic claimed lives till 1960. Most deaths due to the Third Plague Pandemic were reported in Asia, where the pneumonic variant was more prevalent and deadly. Most of the global countries were either British colonies or were indirectly controlled by them. Pandemic did not just take lives, but it also fanned racial inequalities. The plague was started seen as something that is closely associated with cleanliness. The focus shifted to the British's medical aids, and western hygiene practices were the topmost priority. The plague's spread is mainly attributed to transportation and trade between the British and their colonies [33]. The Third Plague Pandemic speeded in different countries as even strict quarantine facilities failed to stop its spread. Different names in various countries know the plague. It is called Manchurian Plague as it gripped the northeastern part of China and some regions of Russia under its clutches. Manchurian Plague of 1910–11 became significant as it was the first plague where medical officers and related officials used cloth masks and personal protective equipment for the first time to contain the airborne plague [32] since similar methods are being used to contain the new coronavirus in 2019–21 (present). The quarantine facilities, physical distancing, closure of non-essential activities, panic-stricken society/human behaviour, and medical aids bear stark similarities [9, 17].

In 1918, as the ashes of the manufactured disaster of World War-I were starting to die down, it was the advent of the deadliest strain in modern times. Influenza virus-infected 40 per cent of the global population over the next 18 months. Out of this 40 per cent, 20 to 50 million people perished due to the influenza virus epidemic. It was massive relative to 17 million people killed in the First World War [5]. The pandemic spread was evident with massive cases in the United States of America and Europe to Greenland.

The Influenza pandemic of 1918–20, also known as the ‘Spanish Flu’, was caused by the H1N1 Influenza A virus. It was Spain that first reported the deaths caused by influenza, and that is how it came to be known as the 'Spanish Flu' [34]. The virus showed a high mortality rate that affected young adults the most. It is considered one of the deadliest pandemics in history. Its temporal frame is parallel to the First World War. It is also known as the 'forgotten pandemic'. Most of the cases were under-reported or went unreported due to the First World War of 1914–1918, which left an indelible mark on the world economy and the grieving families [3]. The Flu's origin is unknown as there are scarce historical and narrative accounts. In her essay *On Being Ill* (1926), Virginia Woolf highlights the need to establish illness as an equal and essential subject of discourse as love and war are.

Molly Schwartz says, *“Despite the fact that the flu claimed 10 times as many American lives as the concurrent world war, it "was hard, first, to characterise a familiar disease like influenza as the enemy," she writes. "The war provided far more compelling enemies, ones that could be seen and put on posters and placed in stories." But the lack of obvious references led to a bit of a myth: the lack of flu art.”* [25, 35]

The nickname ‘Spanish Flu' resulted from a widespread misunderstanding on the political front. Spain was a neutral country with a free press, allowing the media to report on Flu; it was assumed that the Flu originated from Spain, but the Spanish said the virus originated from France, so they called it the 'French Flu.' It reflects on international politics and the blame game in naming any virus, which can be seen in the current context with COVID-19. Different strains of Spanish Flu had a resurgence as Hong Kong flu decades later in 1968 and again as bird flu in 1996, which is still having repercussions globally [36].

Several pandemics' historical and cultural narratives that occurred in the past reflect human behaviour and nature. It is also observed that whenever people's lives were ravaged and devastated by pandemics, humans sought the refuge of religious and medical texts that gave them insight into occurrences of pestilences, and their repercussions were generally mass extinction [31]. Historical narratives aid and fasten the understanding of similar situations so that such health emergencies can be tackled judiciously and certainly.

“*The probability that COVID-19 reaches anything close to the Great Influenza Pandemic seems remote, given advances in public health care and measures that are being taken to mitigate propagation.” They note, however, that some of the mitigation efforts that are currently underway, particularly those affecting commerce and travel, are likely to amplify the virus’s impact on economic activity.”*[37]

The improved medical aids and technological innovations prove significant in monitoring the mortality rate. Catastrophic devastation due to pandemics in history provided insights and helped control potential epidemics to spread as pandemics across the globe. Ebola virus and Zika virus were controlled in specific areas.

Salman Rushdie says, "*As the global pandemic raged, many people failed to take her advice. Voices including an Islamic State spokesman, Hulk Hogan, and a conservative pastor from Florida named Rick Wiles declared that the virus was a punishment from God. Other, greener voices suggested it was nature's revenge on the human race — though, to be fair, louder voices were warning against anthropomorphising”.* [31]

Though there is a marked difference between the first global plague of the sixth century and the new coronavirus of the twenty-first century, human nature and its take on pandemics are common. Science and technology in medicines and drugs have aided in better health conditions and helped tackle the present pandemic to a lower severity.

## Discussion and concluding remarks

### Discussion

It is unbelievable that an invisible and undetectable object could move across the globe and is/was more powerful than humans, states, and empires. It also happens to decimate the sense of security while exposing individuals' vulnerability regimes. This was evident in the nineteenth century with ‘Cholera’ and then in the twentieth century with 'The Spanish Flu'. It brought global differences in the social-economic discrepancy, political hierarchies, and scientific struggles on the surface, which is also reflected in the present conflictive situation.

The world has witnessed several plagues and pandemics in the past. It wreaked havoc and took millions of lives whenever it surfaced on the planet. The recurrent loss of lives, emotional and mental upheavals, economic disbalance, cultural changes, demographic alterations, and societal affliction due to pandemics. In medical emergencies like The Great Plague of 1665, the Cholera pandemic, the Spanish Influenza, or the present coronavirus case, people turn back in history to learn and explore the ways of life. The narratives from the histories of the pandemics give powerful words and stories to live by.

However, pandemics tend to create contradictions, revealing the frictions between openness and closure from one. The COVID-19 pandemic has illustrated this form of behaviour. This is another instance of miniature, accidental mutation in a virus spread into the human population. Nevertheless, the consequences happen to be momentous and disastrous.

The challenge of developing practical barriers against an invisible virus sounds strangely familiar in 2020. However, a centuries-old ongoing debate focused on having a barrier against the agent and its hosts to free others. These debates resonate with behavioural responses to the ongoing COVID-19 pandemic, including the dependence on experts in a time of uncertainty and international organisations but weakened by political hierarchies and power structures. This is also noted in the case of reliance on the WHO to solve a global health crisis and criticism of their efforts.

The most striking feature of this viral agent is proximity and speed in transporting infections. It has undermined the feeling of security and superiority of the global bourgeoisie. For many Europeans, the disease had its place either in the past (e.g., Black Death in Europe) or in less 'civilised' regions. Reflecting on the spatial gap between them and diseases. Nevertheless, with industrialisation, trade, and colonial expansion, fear of worldwide contamination is evident with regular outbreaks and spread across the world with new means of transportation to faraway spaces. This connection and communication have also made a localisable disease a global threat.

Despite commonly shared feelings of humanity, pandemics reveal differences. It is mainly because responses to pandemics are based on imperial hierarchies embedded into the international system and caused discriminative health measures. Consequently, pandemics have acted as a herculean challenge for the policymakers who have constantly been pressured to react judiciously and quickly. During the COVID-19 pandemic, the health emergency left an indelible mark on global politics.

### Findings

There have been several pandemic situations that have eroded millions of lives. In order to trace their markings in the pages of history, the historical narratives of pandemics can be categorised under pre and post-scientific revolution temporal frameworks. Science and technology in health-related issues have played an intrinsic role in controlling and managing pandemic and epidemic-like situations. The physicians and related agencies' approach and perspective toward maladies and pestilence-stricken nations depended on their sources.

The Renaissance era that began in the 1500 and 1600 centuries is known as the enlightenment era. It saw the emergence of modern science where significant scientists and thinkers like Johannes Kepler, Copernicus, and Galileo, gave the initial impetus to exploring science in the human world. William Harvey's contribution to heart and blood movements in the human body was one of the aids of the medical renaissance.

At the beginning of the evolution of science and technology, their contribution helped to dissipate the myths and spiritual beliefs that clerics used to cure diseases. The narratives about pandemics before and after the scientific revolution changed drastically. Science opened paths to see and observe the world as it is.

Managing pandemic is the responsibility of governments. For centuries, city-states in the Italian Peninsula and the Adriatic developed systems tackling plagues in the fourteenth century. While viruses do not respect borders, they spread and survive depending on the effectiveness of laws, policies, and acts of states. The tasks ahead are enormous; ongoing distrust between countries and disagreements is displayed. This has emerged as a significant impediment. Especially when one country's action, or refusal to act, causes risks for other countries and their citizens.

Similarly, evidence suggests that the COVID-19-led health, economic, social, and political crisis reflects the failure of states and institutions. This makes international bodies such as the WHO essential to meet the prest healthcare challenge, but its effectiveness depends on state cooperation. However, powerplay shown by major powers undermined the gravity of the problem and converted the pandemic into a political football. Correspondingly, the capability of the WHO to address pandemics has been questioned. This blame game also happened in the past, when Spain and France blamed each other for the origin and spread of the virus (Spanish Flu) during World War-I. History repeats itself, and the USA blames China for the origin and spread of the COVID-19 virus with frequent use of the terminology 'Chinese virus.'

However, evidence suggests that efficacy in addressing pandemics does not reflect the democratic- authoritarian regimes divide. However, key indicators of successful leadership showcased decision-making, listening to scientists, effective bureaucratic machinery, and the trust they posited in citizens. It is reflected in the ongoing discussion on the performance of women heads of government. Most female leaders made early decisions and took measures with effective communication with the public. Resultantly, these countries suffered relatively low transmission of virus and death rates.

### Conclusion

Over time, the historical narratives of pandemics highlight that human nature and behaviour have defied history. Technological innovations in science and medicines have aided in better health facilities and assisted in battling deadly diseases like AIDS, cancer, neurological, and heart ailments to a certain extent. However, communicable diseases that infect multiple people faster become challenging to tame. Plagues and Flu that infect healthy people faster have proved to be a difficult challenge for scientists, doctors, and most importantly, the common mass. One such evident example is the new coronavirus of the twenty-first century. Though scientists and medical practitioners try their best to procure antidotes for the plagues and Flu, the expected mass plays a vital role in curbing the spread, as seen in past pandemics. Over time, the generation changes and new faces emerge to live history again. It is observed that similar mistakes are repeated by society when met with global medical emergencies and challenges. Society has neglected the seriousness of pandemics till another health challenge is met.

### Limitation of the study

This study is primarily based on a literature review. It largely relied on accessible literature, which could have omitted some relevant studies. Further, the study is limited by the field understanding of pandemic and societal behaviour.

## Data Availability

There is additional data available with the study.
